# Low-level laser therapy in treatment of chemoradiotherapy-induced mucositis in head and neck cancer: results of a randomised, triple blind, multicentre phase III trial

**DOI:** 10.1186/s13014-019-1292-2

**Published:** 2019-05-22

**Authors:** Florence Legouté, René-Jean Bensadoun, Valérie Seegers, Yoann Pointreau, Delphine Caron, Philippe Lang, Alain Prévost, Laurent Martin, Ulrike Schick, Benjamin Morvant, Olivier Capitain, Gilles Calais, Eric Jadaud

**Affiliations:** 1 Département de Radiothérapie, Institut de Cancérologie de l’Ouest - Paul Papin, 15 rue André Boquel, F-49055 Cedex 02, Angers, France; 2 Centre de haute énergie – Oncologie-radiothérapie, 10 boulevard Pasteur, F-06000 Nice, France; 3 Institut de Cancérologie de l’Ouest - Paul Papin, Direction de la Recherche clinique et de l’innovation, 15 rue André Boquel, F-49055 Cedex 02, Angers, France; 4 Centre Jean-Bernard - Clinique Victor-Hugo, 9 rue Beauverger, F-72000 Le Mans, France; 5 Site intégré d’Oncologie - Groupe hospitalier Pitié-Salpétrière, 47-83 boulevard de l’Hôpital, F-75651 Cedex 13, Paris, France; 6 Département de Radiothérapie, Institut Jean Godinot, 1 avenue du Général Koenig, F-51056 Reims, France; 7 Centre de Radiothérapie Guillaume le Conquérant, 61 rue Denfert Rochereau, F-76600 Le Havre, France; 8 Département de Radiothérapie, Centre Hospitalier Universitaire de Brest, Hôpital Morvan, 2 avenue Foch, F-29200 Brest, France; 9 Département de pathologie cellulaire et tissulaire, Centre Hospitalier Universitaire d’Angers, 4 rue Larrey, F-49933, Cedex 09, Angers, France; 10 Département d’Oncologie médicale, Institut de Cancérologie de l’Ouest - Paul Papin, 15 rue André Boquel, F-49055 Cedex 02, Angers, France

**Keywords:** Oral mucositis, Chemoradiotherapy, Head and neck cancer, Lasertherapy, Supportive care

## Abstract

**Background:**

Low-level laser therapy (LLLT) also called Photobiomodulation therapy (PBMT) could reduce oral mucositis (OM) incidence and severity in head and neck cancer patients treated by chemoradiotherapy, however randomised data about efficacy and safety are missing with curative dose 4 J/cm^2^.

**Methods:**

This phase III trial was conducted in patients with oral cavity, or oro/hypopharyngeal cancers (stage III or IV). Patients were treated by lasertherapy on OM lesions grade ≥ 2 (4 J/cm^2^ or placebo), during chemoradiotherapy and until recovery. Severity of OM (incidence and duration of grades ≥3) was used as primary endpoint and blindly assessed.

**Results:**

Among 97 randomised patients, 83 patients (85.6%) could be assessed finally (erroneous inclusions, chemoradiotherapy interruptions) and 32 patients had no lasertherapy because of unreachable OM lesions. Randomisation and population characteristics (sex ratio, age, chemoradiotherapy procedures, toxicities incidence) were still comparable between the two LLLT/PBMT groups. An acute OM (grade ≥ 3) was observed in 41 patients (49.4%): 23 patients (54.8%) of the active laser group versus 18 (43.9%) in the control group (modified intend to treat, *p* = 0.32). Median time before occurrence of OM ≥ grade 3 in half of the patients was 8 weeks in active laser group (vs. 9 weeks in control group). However, 95% of patients exhibited a very good tolerance of LLLT/PBMT.

**Conclusions:**

This study assessed LLLT/PBMT according to the Multinational Association of Supportive care in Cancer recommendations but lacked power. LLLT/PBMT was well tolerated with a good safety profile, which promotes its use in clinical routine for severe OM treatment.

**Trial registration:**

ClinicalTrials.gov Identifier: NCT01772706.

Title: Laser Mucite ORL: Effectiveness of Laser Therapy for Mucositis Induced by a Radio-chemotherapy in Head and Neck Cancer (LaserMucite).

Study Start Date: October 2008.

Primary Completion Date: October 2016.

Responsible Party: Institut de Cancérologie de l’Ouest – Paul Papin.

Principal Investigator: Eric Jadaud, M.D., Institut de Cancérologie de l’Ouest – Paul Papin.

Funding: French Ministry of Health, French national funding scheme (PHRC 2008).

## Background

Oral mucositis (OM) is one of the most common adverse effects of chemotherapy and radiotherapy, especially for head and neck cancer (HNC). Prevalence of iatrogenic mucosal lesions depends on patient characteristics (risk factors) and treatments, particularly with more aggressive approaches: chemotherapy in addition of radiotherapy, or targeted agents as cetuximab during radiation regimen [1, 2].

Several prospective studies specify that all patients reduce their quality of life (QoL) due to oral pain and mucositis; they report these side effects as the most troublesome. In addition, OM is associated with unsatisfactory treatment course (19% of interruptions) and financial burden [3–5]. Moreover unplanned radiation treatment breaks lead to lower outcomes in terms of local control rates [6].

Origin and pathobiology of mucosal damage are still unclear, whereas this side effect is a significant problem for patients and oncologists. OM represents an inflammation of the oral cavity in which mucous membranes are damaged and various lesions are observed: atrophy, erythema, oedema, ulceration, bleeding (National Cancer Institute - Common Terminology Criteria for Adverse Events (NCI - CTCAE) v4.0).

Low-Level Lasertherapy (LLLT) also called Photobiomodulation therapy (PBMT) is a non-invasive care for prevention and management of OM, corresponding to a simple application on mucosa of a high-density monochromatic narrow band light source with various wavelengths (630–830 nm). Many studies showed that LLLT/PBMT during chemotherapy or radiotherapy is effective in OM prevention and treatment [7–16]. Bensadoun’s meta-analysis in 2012, reports eleven randomised placebo-controlled trials with patients treated for HNC, where the relative risk for developing OM could be significantly reduced thanks to LLLT/PBMT, but the used dose should be between 1 to 6 J per point [17]. Another major advantage of LLLT/PBMT relies on the absence of reported in vivo significant toxicity. Nevertheless, the efficacy and the use of LLLT/PBMT are still debated despite the last years growing amount of literature focusing on laser therapy in the mucositis management [18].

Our study evaluates efficacy of low-power laser therapy during concurrent Chemoradiotherapy (CRT) for patients suffering from an advanced HNC: stage III or IV. The strength of this new multicentre phase III is that it focuses on new therapeutic standard: LLLT/PBMT was used in accordance to recent recommendations. This randomised, triple blind clinical trial focuses on observable OM evolution, and secondarily on subjective and functional dimensions of OM (oral soreness, dysphagia, QoL) and possible LLLT/PBMT disadvantages (tolerance, risk of a local relapse).

## Methods

### Objectives

This phase III trial was completed in seven French oncology centres, from October 2008. This multicentre study related HNC patients treated by CRT, likely to develop oral toxicity. Patients included were randomised in a placebo or active treatment arm of lasertherapy. Treatment allocation was centralised, thus randomisation was performed according a 1:1 ratio and stratified by centre.

The main objective was to evaluate the effectiveness of a 100 mW and 658 nm laser, for prevention and treatment of concurrent CRT-induced OM, in advanced oral cavity or oro/hypopharyngeal cancer patients. Primary endpoint was the assessment of LLLT/PBMT efficacy measured by World Health Organization (WHO) grade ≥ 3 OM incidence and duration.

Secondary objectives included: Pain assessment and consumption of painkilling medication, Nutritional state, Cancer treatment compliance, radiotherapy and chemotherapy observance (schedule and interruptions - cause and duration), Laser tolerance, assessed using four-point ratings scales, Quality of life (QoL) with the questionnaire: “EORTC QLQ-H&N35”, Recurrence-Free Survival (RFS), and Overall survival (OS): calculated from randomisation date until patient’s death for a 5-year period.

### Patients

Patients were recruited from seven French centres (departments of medical oncology and radiotherapy): Integrated Centre for Oncology – Paul Papin (Angers), Poitiers University Hospital, Pitié-Salpétrière University Hospital (Paris), Specialist centre for cancer care – Jean Godinot (Reims), The Armoricaine Clinic (Saint-Brieuc), Guillaume le Conquérant radiotherapy centre (Le Havre) and Brest University Hospital. To be enrolled, they should exhibit a locally advanced histologically proven squamous cell carcinoma of oral cavity, oropharynx or hypopharynx (stage III or IV). Subjects had a scheduled CRT (without or after surgery), with platinum salts (with or without 5-FU) or cetuximab alone. The other inclusion criteria were: between 18 and 75 years of age, performance status (WHO score) ≤ 2, life expectancy ≥3 months without cancer treatment, serum creatinine < 150 μmol/L and creatinine clearance ≥55 mL/min (Cockcroft-Gault formula), blood cell count: haemoglobin > 8 g/dL, neutrophils > 1500 /mm^3^, platelets > 100,000/mm^3^, hepatic transaminases (AST and ALT) and alkaline phosphatase < 2,5 N, total bilirubin < 1,5 N, and reliable contraceptive method among women of child-bearing age.

The exclusion criteria were neo-adjuvant chemotherapy; distant metastasis; previous malignancy over the last 5 years (except basal cell carcinoma or carcinoma in situ of the uterine cervix); previous radiotherapy in the head and neck region; severe allergy to platinum salts; any uncontrolled comorbidity (pulmonary, kidney, liver, or heart failure); pregnancy or breastfeeding.

### Treatments

Every patient was randomly assigned to active laser group (group A) or control group with placebo (group B). LLLT/PBMT was based on a He-Ne laser HETSCHL® (lambda = 658 nm, output = 100 mW and energy density = 4 J/cm^2^). All the patients received same instructions about oral hygiene and abstinence from tobacco and alcohol. LLLT/PMBT began at first visible signs of grade OM lesions. All anatomic sites with moderate or severe OM (OMS scale grade ≥ 2) were daily treated after radiotherapy session, according recommendation: 40 s per site of 1cm^2^ to reach 4 J/cm^2^. Macroscopic involved oral area (tumour site) was excluded from LLLT/PBMT applications. Protective eyeglasses were used to avoid detrimental effects of the laser beam on eyes, and thus blind procedure was respected. The differences during laser session were laser-on or laser-off condition and time applications, known to the physician, not to the patient. LLLT/PBMT was interrupted when OM was less acute than grade 2. The Table 1 summarises lasertherapy parameters.

**Table 1:** Lasertherapy/photobiomodulation therapy parameters

	Arm A Active Laser group	Arm B Placebo Laser group
Device	He-Ne laser HETSCHL®	
Wavelength	Lambda = 658 nm	
Power	100 mW	
Device condition	ON	OFF
Irradiation time	40 s/cm^2^	10 s/cm^2^
Energy density	4 J/cm^2^	0 J/cm^2^
Dose administrated	4 J	0 J
Beam area	Intraorally: 1 cm^2^ per application	
Pulse parameter	Pulsed < 50 Hz	
Anatomical Location	Oral mucositis (Lips - Mouth – Oropharynx) Tumour location was excluded from treated area Maximum area: 10 cm^2^	
Number of treatments	1 session / day, 5 times / week From day of grade II OM occurrence to day of grade II OM resolution	
Interval between treatments	1 or 2 days (treatments on radiotherapy days).	

All patients received radiation treatment: conformational technique or Intensity-Modulated Radiation Therapy (IMRT) according the same modalities (2.0 Gy/day, 5 sessions/week). Dose was prescribed according to the surgical option: adjuvant or exclusive CRT and constraints for critical normal tissue structures were respected. Treatment consisted in one Gross tumour volume (GTV) and two Clinical target volumes (CTV): “low risk CTV” and “high risk CTV” with a dose of 50 Gy or 70 Gy. In the absence of GTV, “low risk ¨PTV” received 50 Gy and “high risk PTV” received 60 to 66 Gy. Planning target volumes (PTV) were defined thanks to a margin of 0.5 cm around CTV. Volume delineation were established according consensus guidelines [19].

Concomitant radiosensitising chemotherapy was delivered, according to surgical statement and to patient’s comorbidities: Cisplatin with 5-Fluorouracil, Cisplatin alone, Carboplatin with 5-Fluorouracil or Cetuximab alone.

### Assessments and statistical analysis

Assessments of primary and secondary outcomes were triple blind (patients, investigators and statisticians). Physician who treated a patient did not participate to patient’s clinical outcomes assessment. Patients were weekly assessed for mucositis during CRT schedule and until OM healing. Next, cancer surveillance was led for 5 years. Evaluations were undertaken every 3 months the 1st year and then every 6 months. Examination data were recorded in forms specially designed for this study: OM was graded from 0 to 4, using WHO criteria. A numeric pain rating scale (graded from 0 to 10) was used to evaluate pain and patients had to fill another form to express their QoL during cancer treatments.

To detect LLLT/PBMT effectiveness, the following hypothesis was tested: LLLT/PBMT would lead to a decrease of 30% in the severe OM grade incidence. Indeed, literature reported severe OM for almost 67% of patients treated for locally advanced HNC. The alpha risk was set at a standard level of 5% and beta level of 20% (study power of 80%), and hence 43 subjects had to be included for each treatment arm. With 15% of not assessable patients (loss of follow-up, protocol deviations), 100 patients had to be enrolled in order to demonstrate a significant difference compared to placebo. Randomisation with ratio 1:1 was stratified by centre. Patient data was analysed in modified intention to treat, and then with per-protocol statistics.

To fulfil the main objective, severe OM rate (grade ≥ 3) was estimated in the two treatment arms with its confidence interval, and Chi-Square test was conducted for comparison. For secondary outcomes (pain, nutritional statement, treatment compliance, QoL, laser tolerance), a Mann-Whitney test was used for quantitative parameters and a Chi-Square test for qualitative data. Survival data, duration of severe OM and delay between the emergence of severe OM and initiation of treatment were analysed by Kaplan Meier method. Comparisons were done thanks to log-rank test. All statistical tests were bilateral and statistical significance rate in terms of the results was set at a level of 5% (*p* ≤ 0.05). Statistical analyses were done using R software (version: 3.3.2).

### Ethics and research funding

This trial was funded thanks to a National public grant (PHRC 2008) and complied with the Declaration of Helsinki and European directive about clinical trials (2001/20/CE). This study was registered on ClinicalTrials.gov (number: NCT01772706).

## Results

### Population

Ninety-seven patients were enrolled from October 2008 to March 2016 and were randomised into active lasertherapy group or placebo-controlled group. The study will end 5 years after the last inclusion (March 2021).

Eighty-three patients were assessed: 42 patients randomised in active lasertherapy arm and 41 patients in placebo arm. Among exclusion criteria, were found no radiation or incomplete radiotherapy (< 30 Gy), no chemotherapy administrated, OMS Performans status ≥3, inappropriate histology of diagnosed cancer, previous malignancy (lung cancer).

**Fig. 1:**
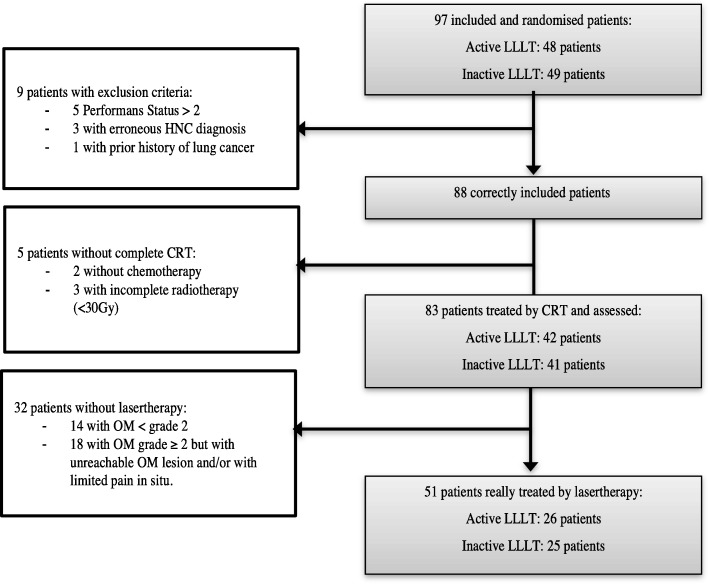
Flowchart. CRT: Chemoradiotherapy, LLLT: Low-Level Laser Therapy, OM: Oral Mucositis, Reasons of exclusion: - Patients without complete chemoradiotherapy, - Patients with exclusion criteria. Randomisation was still well balanced despite the exclusion of 14 patients. Only 51 patients were treated by lasertherapy (active or inactive): no imbalance was observed between the LLLT groups

Overall, after secondary exclusions, 83 patients could be assessed for the oral injured mucosa. Flowchart and reasons of exclusion were presented in Fig. 1. Patients’ characteristics were the same in both groups and randomisation was still well balanced despite excluded patients (Tables 2 and 3).

**Table 2:** Population characteristics

		Overall	Arm A (active LLLT/PBMT) (*N* = 42)	ArmB (placebo LLLT/PBMT) (*N* = 41)	*p*-value
Gender	Female	8 (9.6%)	5 (11.9%)	3 (7.3%)	0.74
	Male	75 (90.4%)	37 (88.1%)	38 (92.7%)	
Age (years)	Mean ± SD	58 (53–65)	58 (53–62)	58 (53–68)	0.25
Tumour Location	Oral cavity	17 (20.5%)	9 (21.4%)	8 (19.5%)	0.70
	Hypopharynx	19 (22.9%)	11 (26.2%)	8 (19.5%)	
	Oropharynx	47 (56.6%)	22 (52.4%)	25 (61.0%)	
TNM classification	T1	7	3 (7.1%)	4 (9.8%)	0.09
	T2	24	16 (38.1%)	8 (19.5%)	
	T3	34	16 (38.1%)	18 (43.9%)	
	T4a	13	7 (16.7%)	6 (14.6%)	
	T4b	5	0 (0%)	5 (12.2%)	
	N0	15	5 (11.9%)	10 (24.4%)	0.36
	N1	6	2 (4.8%)	4 (9.8%)	
	N2	56	31 (73.8%)	25 (61.0%)	
	N3	5	3 (7.1%)	2 (4.9%)	
	Nx	1	1 (2.4%)	0 (0%)	
	M0	83 (100%)	42 (100%)	41 (100%)	
Histologic differentiation	Well-differentiated	36 (43.4%)	17 (40.5%)	19 (46.3%)	0.25
	Unknown	6 (7.2%)	4 (9.5%)	2 (4.9%)	
	Moderately differentiated	31 (37.3%)	18 (42.9%)	13 (31.7%)	
	Poorly differentiated	10 (12.0%)	3 (7.1%)	7 (17.1%)	
Smoking status	Never-smokers	12 (14.5%)	3 (7.1%)	9 (22%)	0.11
	Smokers	71 (85.5%)	39 (92.9%)	32 (78%)	
During CRT	Smoking cessation	8 (11.3%)	4 (10.3%)	4 (12.5%)	0.95
	Active smokers	23 (32.4%)	13 (33.3%)	10 (31.3%)	
	Unspecified	40 (56.3%)	22 (56.4%)	18 (56.3%)	
Alcohol consumption	No	22 (26.5%)	11 (26.2%)	11 (26.8%)	0.92
	Unknown	3 (3.6%)	1 (2.4%)	2 (4.9%)	
	Yes	58 (69.9%)	30 (71.4%)	28 (68.3%)	
During CRT	Alcohol withdrawal	8 (13.8%)	3 (10.0%)	5 (17.9%)	0.72
	Active alcohol use	21 (36.2%)	11 (36.7%)	10 (35.7%)	
	Unspecified	29 (50.0%)	16 (53.3%)	13 (46.4%)	
Feeding	Enteral feeding	9 (10.8%)	3 (7.1%)	6 (14.6%)	0.39
	Liquid diet	1 (1.2%)	1 (2.4%)	0 (0%)	(0.64 with binary data)
	Minced food	23 (27.7%)	13 (31%)	10 (24.4%)	
	Unspecified	2 (2.4%)	0 (0%)	2 (4.9%)	
	Solid diet	48 (57.8%)	25 (59.5%)	23 (56.1%)	

*TNM* Tumour/Node/Metastasis*.*

*CRT* Chemoradiotherapy.

**Table 3:** Comparison of patient characteristics

		No LLLT/PBMT (*n* = 32)	LLLT/PBMT (active or placebo) (*n* = 51)	*p*-value
Gender	Female	6 (18.8%)	2 (3.9%)	0.05006
	Male	26 (81.3%)	49 (96.1%)	
Age (years)	Mean ± SD	57.5 (52.7–64.5)	59 (53–65)	0.97
Tumour Location	Oral cavity	8 (25%)	9 (17.6%)	0.60
	Hypopharynx	8 (25.0%)	11 (21.6%)	
	Oropharynx	16 (50.0%)	31 (60.8%)	
TNM classification	T1	5 (15.6%)	2 (3.9%)	0.20
	T2	8 (25.0%)	16 (31.4%)	
	T3	10 (31.2%)	24 (47.1%)	
	T4a	7 (21.9%)	6 (11.8%)	
	T4b	2 (6.2%)	3 (5.9%)	
	N0	3 (9.4%)	12 (23.5%)	0.25
	N1	3 (9.4%)	3 (5.9%)	
	N2	24 (75.0%)	32 (62.7%)	
	N3	1 (3.1%)	4 (7.8%)	
	Nx	1 (3.1%)	0 (0%)	
Histologic differentiation	Well-differentiated	15 (46.9%)	21 (41.2%)	0.95
	Unknown	2 (6.3%)	4 (7.8%)	
	Moderately differentiated	12 (37.5%)	19 (37.3%)	
	Poorly differentiated	3 (9.4%)	7 (13.7%)	
Smoking status	Never-smokers	4 (12.5%)	8 (15.7%)	0.94
	Smokers	28 (87.5%)	43 (84.3%)	
During CRT	Smoking cessation	1 (7.7%)	7 (38.9%)	0.095
	Active smokers	12 (92.3%)	11 (61.1%)	
Alcohol consumption	No	9 (28.1%)	13 (25.5%)	0.64
	Unknown	2 (6.3%)	1 (2.0%)	
	Yes	21 (65.6%)	37 (72.5%)	
During CRT	Alcohol withdrawal	3 (33.3%)	5 (25.0%)	0.99
	Active alcohol use	6 (66.7%)	15 (75.0%)	
Feeding	Enteral feeding	4 (12.5%)	5 (9.8%)	0.84
	Liquid diet	0 (0%)	1 (2.0%)	
	Minced food	7 (21.9%)	16 (31.4%)	(binary
	Unspecified	2 (6.3%)	0 (0%)	data)
	Solid diet	19 (59.4%)	29 (56.9%)	

*TNM* Tumour/Node/Metastasis*.*

*CRT* Chemoradiotherapy*.*

Enrolment started slowly with only 23 patients included at the end of 2010, and six patients among them were erroneously included. Among the 83 patients correctly included in this trial, 54 patients (65.1%) were treated in the main investigator centre: the Integrated Centre for Oncology, Angers.

### Primary endpoint: oral mucositis

For the final analysis, only 83 patients (Table 2) were assessed because 14 patients had exclusion crtieria. Therefore, oral mucositis was assessed in modified intention to treat on 83 subjects. Among these 83 patients, severe and reachable OM occurred only in 51 patients. Lasertherapy (active or placebo) was conducted only for 51 subjects, that is why a per protocol analysis was also performed.

**Fig. 2:**
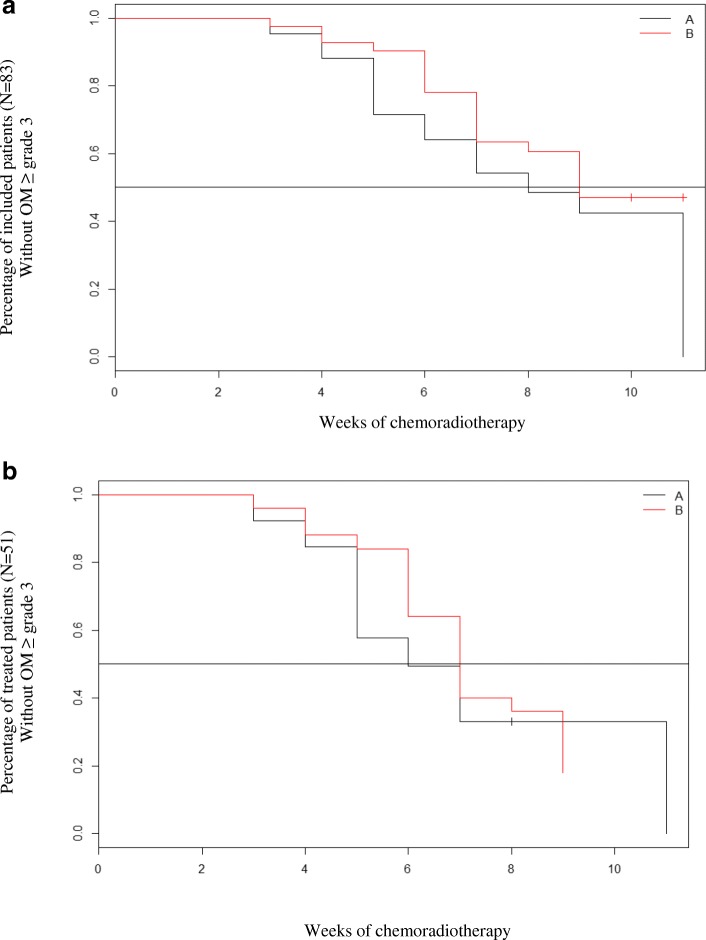
**a**: Time to first occurrence of OM grade ≥ 3 – Modified intention to treat analysis. **b**: Time to first occurrence of OM grade ≥ 3 – Per protocol analysis. *Arm A: active Lasertherapy arm - Arm B: control placebo, arm (Laser-off treatment)*, *OM: Oral mucositis*

Forty-one patients suffered from OM ≥ grade 3 during CRT: 23 patients (54.8%) in active LLLT/PBMT arm versus 18 patients (43.9%) in placebo arm. No statistically significant difference (NS) was observed (*p* = 0.32). The median time to first occurrence of OM with a grade ≥ 3 is 8 weeks in the active LLLT/PBMT arm versus 9 weeks in the placebo arm, without statistically significant difference (log rank *p* = 0.22). Comparison of times to first occurrence of OM ≥ grade 3 is presented in Fig. 2a. Sixty-eight patients (81.9%) suffered from OM ≥ grade 2 (without difference between the two arms), and 63.2% of them kept lesions of OM grade ≥ 2 at the end of CRT. The median time to first occurrence of OM with a grade ≥ 2 is 5 weeks in the active LLLT/PBMT arm versus 6 weeks in the placebo arm (p: NS).

Among 83 included patients: 32 patients were not eligible for LLLT/PBMT (unreachable lesions or OM ≤ grade 1); hence, 51 patients (61.4%) could be assessed after LLLT/PBMT: 36 patients (70,6%) suffered from OM ≥ grade 3: 18 patients (62.2%) in active LLLT/PBMT arm versus 18 patients (72.0%) in placebo arm (NS: *p* = 0.83). The median time to first occurrence of OM with a grade ≥ 3 is 6 weeks in the active LLLT/PBMT arm versus 7 weeks in the placebo arm (NS, log rank *p* = 0.61, Fig. 2b).

### Secondary endpoints

#### Nutritional status

When radiotherapy ended, 54.1% of patients presented a weight loss of more than 5 and 17.6% had a weight loss of more than 10%. There was no significant difference between the two arms, nor, if patients continued lasertherapy after completion of CRT. Feeding data of 68 patients (81.9%) were available at the beginning of CRT: 62 patients (91.2%) had a normal diet or, at least, could eat solid food. At the end of CRT, 37 patients (59.7%) moved to liquid diet or enteral feeding. There was no difference between the two LLLT/PBMT groups for nutritional assessment (*p* = 0.39).

#### Pain

**Fig. 3:**
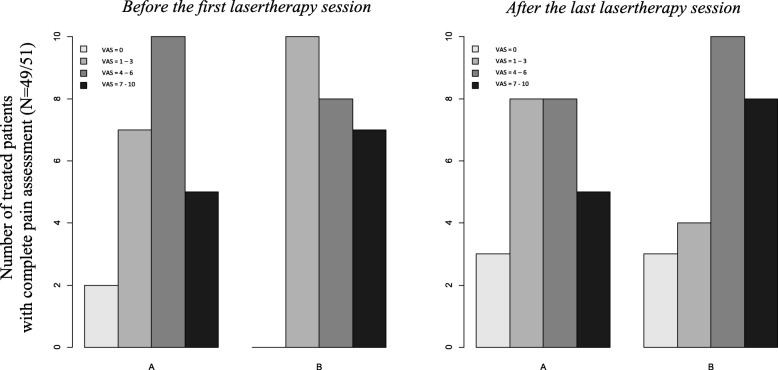
Pain assesment – at the first and at the last lasertherapy session - Per protocol analysis. *Arm A: active Lasertherapy arm - Arm B: control placebo arm (Laser-off treatment)*, VAS: Visual Analogue Scale: A numeric pain rating scale was used (0: ‘No pain’ to 10: ‘Worst pain possible’)

At the end of CRT, 77 patients (92.8%) had an available pain evaluation: 42.8% had a moderate or severe pain score. During CRT, 69 patients (83.1%) took painkillers: 33 in active LLLT/PBMT arm vs. 36 in placebo arm (NS, *p* = 0.41). Forty-five patients used major analgesics: 21 in active LLLT/PBMT arm vs. 24 (NS, *p* = 0.58). Among 51 patients treated by lasertherapy, two patients from arm A had incomplete pain assessment (missing data during lasertherapy weeks). From 49 validated pain scale assessments, there was no analgesia difference in per-protocol analysis (NS, *p* = 0.27, Fig. 3).

#### Quality of life (QoL)

Multi-scale questionnaires assessed weekly quality of life of 50 patients who underwent lasertherapy with OM ≥ grade 2. There was no difference between the two LLLT/PBMT arms for 17 dimensions, one dimension (sticky saliva) was in favour of placebo arm (*p* = 0.004): 4 patients (16%) vs. 15 patients (60%). However, this difference was not confirmed by the ‘swallowing’ dimension, nor by the ‘dry mouth’ dimension, thus these results had to be interpreted with caution.

#### CRT compliance

Among 88 patients (randomised and correctly included), five patients were secondarily excluded because they did not achieve prescribed CRT (one death and one surgical complication, unrelated to the laser treatment) or had a chemotherapy regimen other than those allowed. There was no statistically significant difference in chemotherapy regimens between the two arms of LLLT/PBMT. Most patients received three chemotherapy cycles (median number of cycles): 15.7% of patients were treated by Carboplatin and 5-FU, 33.7% Cisplatin and 5-FU, 44.6% Cisplatin alone, 4.8% of patients: Carboplatin alone, 7.2% Cetuximab and 6.5% several chemotherapy regimens. There was no statistically significant difference in radiotherapy regimens between the two arms of LLLT/PBMT, 74.7% of patients benefited from IMRT. Patients received 35 fractions (mean ± SD: 34 [32–36]) and 8 weeks of treatments (mean ± SD: 8 weeks [8, 9]). Median delivered doses were: Low risk PTV = 56.0 Gy (mean ± SD: 55.9 ± 5.8) and high risk PTV = 69.9 Gy (mean ± SD: 68.3 ± 3.0).

Among 83 patients: 18.1% had almost a delay explained by radiotherapy-induced toxicities (no difference between the two laser arms). Many other reasons of delay (299 sessions) were found: maintenance of radiotherapy equipment: 72 patients (122 sessions), public holidays: 65 patients (114 sessions), severe toxicity: 15 patients (20 sessions).

#### Lasertherapy: treatment compliance and tolerance

Among 83 analysed patients: 14 patients did not show any OM ≥ grade 2 and were not treated by LLLT/PBMT, 18 patients had an OM ≥ grade 2 but did not receive LLLT/PBMT due to unreachable OM lesions, limited pain in situ, or CRT compliance issue. Finally, 51 patients (61.4%) were treated by LLLT/PBMT (active or placebo). Tolerance was excellent for every session for 91% of patients and 4.5% in most sessions, and only 4.5% had a moderate level of tolerance for several sessions. There was no difference between active LLLT and placebo arms about modalities of LLLT/PBMT (number of sessions, surface of laser treatment).

Thirty-five patients presented OM lesions ≥ grade 2 at the end of CRT and 20 patients among them (57.1%) agreed to continue LLLT/PBMT with an excellent tolerance: 12 with active LLLT/PBMT vs. 9 with placebo (NS).

#### Locoregional control and survival

No statistical analysis was undertaken yet, because of many missing data. The follow-up period was planned for 5 years, and more than 50% of patients were included from 2013. Neither severe adverse effect of LLLT/PBMT, nor unexpected death due to lasertherapy was reported. This survival data will be up-dated in 2021.

## Discussion

LLLT/PBMT is used since the 1960s for many clinical objectives and presents several advantages. First of all, the procedure does not generate pain or heat, because only a low energy is transferred to the tissues by photobiomodulation. Nowadays LLLT is rather named PBMT as recommended by the World Organization for Laser Therapy (WALT). There are substantial data to affirm that PBMT/LLLT can impact favourably incidence and severity of OM, especially for HNC and hematologic malignancies [20, 21]. Migliorati’s systematic review of LLLT/PBMT in management of OM for cancer patients, presents two new guidelines since 2013, confirmed by European Society for Medical Oncology (ESMO) clinical practice guidelines for oral mucosal injury [22]. There are strong evidences for LLLT/PBMT in prevention of OM in patients receiving hematopoietic stem cell transplantation and with high-dose chemotherapy conditioning. LLLT/PBMT was suggested for the prevention of OM in HNC patients who undergo radiotherapy. Other validated treatment options for OM management are relatively rare and still palliative: mouth baths and analgesics.

According Sonis et al., it is necessary to keep in mind that LLLT/PBMT led to several biological effects exceeding the ones observed with traditional therapies [23]. LLLT/PBMT is also used in many benign conditions, this is why it is still sometimes considered as ‘an alternative therapy’ and underestimated. Thus, this kind of application should not be compared to LLLT/PBMT application in cancer patient, in whom challenges and issues are radically different. Several authors even suggest that LLLT/PBMT could maybe enhance the malignant potential of the primary tumour (fostering local growth and invasion). Unfortunately, literature is contradictory on this point [23, 24]. In light of those facts, a precautionary principle must be discussed. The question is whether this cancer supportive care may help or jeopardise cancer treatment. Data on LLLT/PBMT biological effects demonstrate an action on tumour behaviour, in addition of a positive impact of oral mucositis and cancer treatment tolerance. That explains why the application of LLLT/PBMT must avoid tissue within the tumour field and why time and energy application must be strictly controlled, as recommended in our study [4, 17]. An extreme scientific rigour is requested for pharmacological interventions in oncology studies, whereas a lack of consistent harmonisation about LLLT/PBMT parameters is found in clinical trials. A potential negative impact on tumour behaviour and treatment responsiveness encourages further investigations, with robust methodology and a long follow-up to assess relapse risk and overall survival [25]. Physicians have to be careful during laser application, due to the probable narrow therapeutic index of LLLT/PBMT. The idea of benefit-risk ratio is tightly linked to LLLT/PBMT parameters.

In order to personalise treatment, several subgroups of patients could be highlighted with different individual response profiles. Thanks to well-designed prospective controlled studies, different protocols of LLLT/PBMT could be analysed [26]. In France, almost fifteen oncology centres regularly use soft laser and about half of these had included patients in our trial. Slow progress of inclusions, data missing and attrition bias may be considered as key difficulties of our study. Difficulties to include subjects in a supportive care study are explained by lots of therapeutic trials with new antineoplastic treatments proposed for locally advanced HNC. Our study assessed supportive care, a main question in care, but often physicians have preferred to enroll patients in surgery, chemo or radiotherapy trials. Moreover, many HNC cancer patients have declined any inclusion in a study protocol. Inclusion and exclusion criteria were hard to obtain: mucositis grade II at the inclusion needed a pre-enrolment before the toxicities occurrence.

Triple blind evaluation: for patient, assessor and statistician avoids an assessment bias. This methodology is found in many studies and it is a preferable option for every phase III trial, but it implies to strictly organise patient pathway and more medical and paramedical time [7, 13, 15, 27–29]. Our trial proposed LLLT/PBMT when OM was greater than grade 1 and interrupted when it was lower than grade 2, in order to spare medical time and focusing on severe mucositis as main issue. Data missing and attrition bias are perhaps linked to violation protocol with inappropriate delegation to not well-trained paramedical staff. Blind procedure requires more human resources: one person to treat and another to assess clinical issues. It is possible that some centres cannot furnish optimal organisational capacities: they finally include few patients and have to cope with laser administration errors. If laser application should be delegated in a trial or even in clinical routine practice, a specific medical prescription and an appropriate training would be needed for nurses or medical radiation technologists [30].

Another heterogeneity source in our trial population was the development of new treatments during patients’ inclusion period: IMRT for radiotherapeutic techniques and cetuximab as a new-targeted therapy. Those advancements in cancer treatment do not reduce incidence of high OM despite a potential reduction of oral mucosa volume exposed to high dose (≥ 30 Gy). Moreover, cetuximab-treated patients often experience significant more severe OM, but without necessarily lower QoL [31–34].

Lastly, investigators should strictly use clinical mucositis assessment scales. WHO scale has the advantage to focus on mucosal ulceration and feeding capacity. The inter-evaluator variability is also found in OM scoring, thus assessment standardisation and training are essential. The severity of mucosal damage must be objectively classified [25, 35, 36]. Some reports suggest a good concordance between self-reported questionnaire and physician measures of OM severity, but patients are able to detect symptoms changes earlier than clinicians are. In any case, a QoL questionnaire could avoid underreporting of OM, in particular when clinical examinations fail to correctly scoring OM or mouth/throat soreness [37, 38].

Many recommendations were published thanks to the Multinational Association of Supportive care in Cancer (MASCC) after the study started [20]. There are several other options to treat oral mucositis. For instance, honey and other oral topics would be an interesting option for mucositis treatment [39]. Furthermore, it would have been interesting to compare lasertherapy to these local treatments, or to use them in association.

New studies should be designed with a special attention about blind conditions, number of patients included, randomisation time and monitoring, laser procedure. For instance, like our study, other trials found negative results about severe OM reduction, without excluding a marginal benefit in CRT tolerance thanks to LLLT/PBMT [29]. In our study, only half of randomised patients could really benefit from LLLT and be assessed because of unreachable OM lesions (into the oropharynx and hypopharynx), that implies a potential lack of statistical power. Less stringent laser technologies should be considered for further trials: dedicated device self-treatment or cutaneous and OM concurrent treatment with extra oral applicators.

## Conclusions

While many adverse drug reactions may be declining, mucosal damage remains an area of concern for HNC treatment. This randomised multicentre phase III trial was designed to evaluate lasertherapy (photobiomodulation therapy) as a supportive care in oral mucositis management for HNC patients, according to the MASCC recommendations with curative dose of 4 J/cm^2^. LLLT/PBMT was well tolerated with a good safety profile for treated patients. Despite this encouraging data, this study lacks power. Other largest phase III trials are needed to improve LLLT/PBMT procedures.

## Abbreviations

CRT: Chemoradiotherapy (Chemoradiation)
CTV: Clinical Target Volume
EORTC: European Organisation for Research and Treatment of Cancer
ESMO: European Society for Medical Oncology
GTV: Gross Tumour Volume
HNC: Head and Neck Cancer
IMRT: Intensity-Modulated Radiation Therapy
ISOO: International Society of Oral Oncology
LED: Light-Emitting Diode
LLLT: Low-Level Laser Therapy
MASCC: Mucositis Study Group of the Multinational Association of Supportive Care in Cancer
NCI-CTC: National Cancer Institute – Common Toxicity Criteria
NS: Non-Significant
OM: Oral Mucositis
ORL: Otorhinolaryngology
OS: Overall Survival
PBMT: Photobiomodulation therapy
PHRC: National Public Grant (Programme Hospitalier de Recherche Clinique)
PTV: Planning Target Volume
QoL: Quality of Life
QoLH&N: Quality of Life questionnaire
RFS: Recurrence Free Survival
RTOG: Radiation Therapy Oncology Group
TNM: Tumour / Node / Metastasis
VAS: Visual Analogue Scale
WALT: World Organization for Laser Therapy
WHO: World Health Organization
3D: 3 Dimensions
5-FU: 5-Fluorouracil
